# Robot enhanced stroke therapy optimizes rehabilitation (RESTORE): a pilot study

**DOI:** 10.1186/s12984-021-00804-8

**Published:** 2021-01-21

**Authors:** Alexa B. Keeling, Mark Piitz, Jennifer A. Semrau, Michael D. Hill, Stephen H. Scott, Sean P. Dukelow

**Affiliations:** 1 Hotchkiss Brain Institute, University of Calgary, Calgary, AB Canada; 2 Department of Clinical Neurosciences, University of Calgary, Calgary, AB Canada; 3 Department of Kinesiology and Applied Physiology, University of Delaware, Newark, DE USA; 4 Department of Biomedical and Molecular Sciences, Queen’s University, Kingston, ON Canada

**Keywords:** Stroke rehabilitation, Robotic rehabilitation, Robotics, Subacute stroke

## Abstract

**Background:**

Robotic rehabilitation after stroke provides the potential to increase and carefully control dosage of therapy. Only a small number of studies, however, have examined robotic therapy in the first few weeks post-stroke. In this study we designed robotic upper extremity therapy tasks for the bilateral Kinarm Exoskeleton Lab and piloted them in individuals with subacute stroke. Pilot testing was focused mainly on the feasibility of implementing these new tasks, although we recorded a number of standardized outcome measures before and after training.

**Methods:**

Our team developed 9 robotic therapy tasks to incorporate feedback, intensity, challenge, and subject engagement as well as addressing both unimanual and bimanual arm activities. Subacute stroke participants were assigned to a robotic therapy (N = 9) or control group (N = 10) in a matched-group manner. The robotic therapy group completed 1-h of robotic therapy per day for 10 days in addition to standard therapy. The control group participated only in standard of care therapy. Clinical and robotic assessments were completed prior to and following the intervention. Clinical assessments included the Fugl-Meyer Assessment of Upper Extremity (FMA UE), Action Research Arm Test (ARAT) and Functional Independence Measure (FIM). Robotic assessments of upper limb sensorimotor function included a Visually Guided Reaching task and an Arm Position Matching task, among others. Paired sample t-tests were used to compare initial and final robotic therapy scores as well as pre- and post-clinical and robotic assessments.

**Results:**

Participants with subacute stroke (39.8 days post-stroke) completed the pilot study. Minimal adverse events occurred during the intervention and adding 1 h of robotic therapy was feasible. Clinical and robotic scores did not significantly differ between groups at baseline. Scores on the FMA UE, ARAT, FIM, and Visually Guided Reaching improved significantly in the robotic therapy group following completion of the robotic intervention. However, only FIM and Arm Position Match improved over the same time in the control group.

**Conclusions:**

The Kinarm therapy tasks have the potential to improve outcomes in subacute stroke. Future studies are necessary to quantify the benefits of this robot-based therapy in a larger cohort.

*Trial registration:* ClinicalTrials.gov, NCT04201613, Registered 17 December 2019—Retrospectively Registered, https://clinicaltrials.gov/ct2/show/NCT04201613.

## Background

The vast majority of clinical trials in stroke rehabilitation have focused on individuals with chronic stroke [1]. While therapy delivered in the chronic phase after stroke has been shown to lead to reductions in motor impairments [2–4], a significant opportunity for improvement in motor abilities appears to occur during the subacute phase [5]. During this time, we tend to see the most rapid recovery of motor abilities, and some studies have suggested that more intensive intervention in subacute stroke can enhance the return of motor abilities [6–9]. The present manuscript focuses on the development and pilot testing of robotic tools targeted at augmenting recovery in subacute stroke.

The first robot-based therapy studies for the post-stroke upper extremity began to appear in the late 1990s [10–12]. Robotics have the potential to measure and increase the number of movement repetitions an individual performs in a given time period compared to conventional therapy, and some have speculated that this should lead to improved recovery [13–16]. Robotic therapy may also provide a way of increasing the dose of therapy without necessarily requiring more therapists. Verbeek et al. (2017) noted in a recent systematic review that robotic therapy produced a significant, although small, effect on improving motor control and muscle strength, but evidence in the first 3 months after stroke was lacking [17]. This review brought to light the variety of rehabilitation robotics used for stroke recovery with two main classifications for upper-extremity robotics: end-effector and exoskeleton. End effector devices are usually simpler, only directly interacting with the most distal parts of the participant whereas exoskeleton devices can align with one or many joints allowing for direct measurement and manipulation of joint movement [18]. These robot types can be further divided into unimanual or bimanual, active or passive, and planar or 3-dimensional [19].

**Fig. 1:**
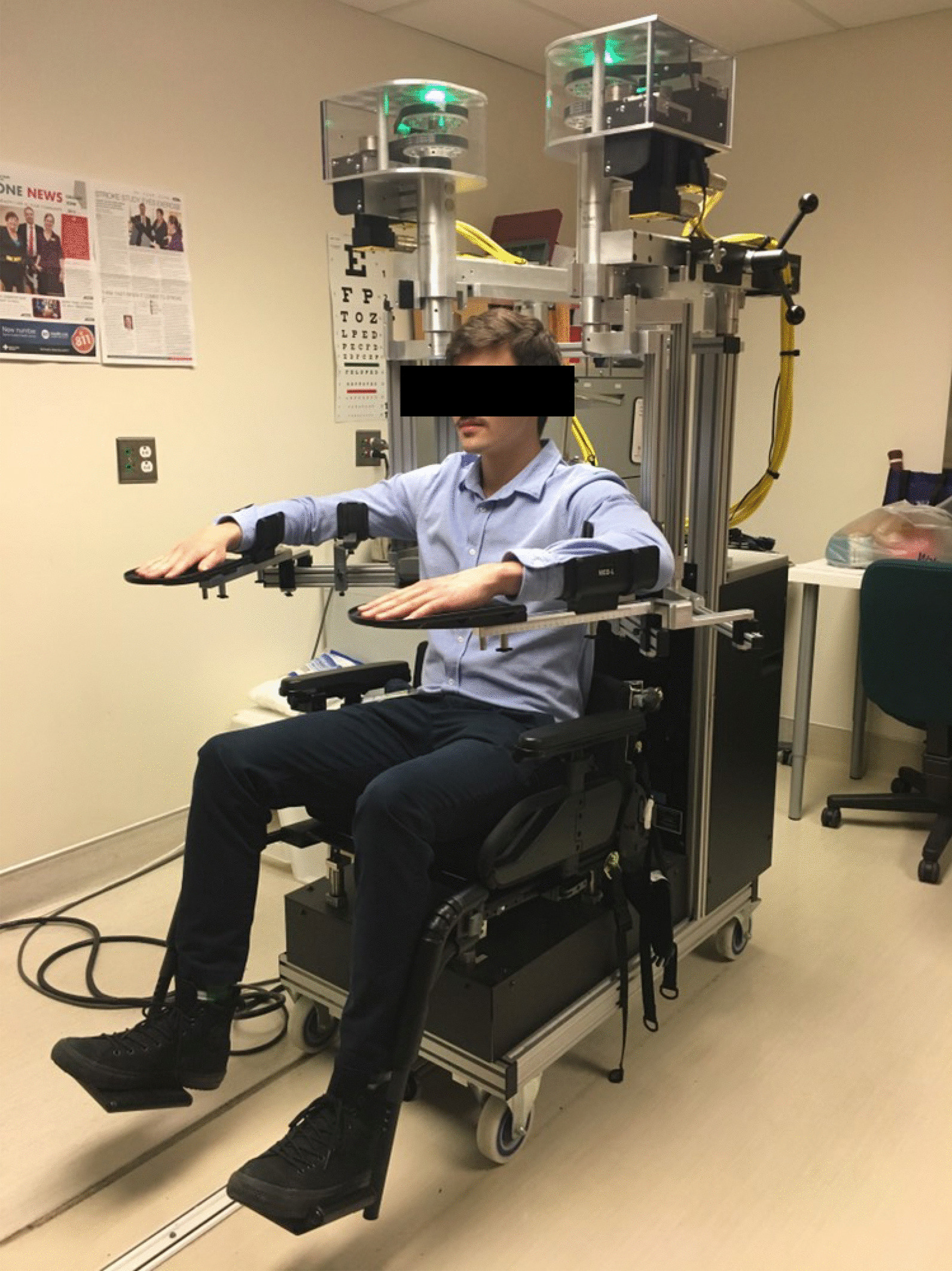
The Kinarm Exoskeleton. A frontal view of the Kinarm Exoskeleton

Our group has significant experience with a device called Kinarm (Fig. 1). It is a planar, bimanual, multi-joint robot paired with a virtual reality display [20] (Kinarm, Kingston, Ontario). To date, studies using the Kinarm exoskeleton have focused on its validity as an assessment tool for sensation, motor function, and cognition after stroke [21–26], transient ischemic attack [27], brain injury [28, 29], healthy aging [30], and many other diagnoses. Such multi-domain assessments take ~ 45 min and have been well tolerated by individuals who are only a few days post-stroke. In addition, the bimanual support the device can provide to the limbs allows for the possibility of bimanual interaction [31], which has been suggested to be a missing piece in current stroke rehabilitation programs [32, 33]. This would allow us to develop rehabilitation tasks that were engaging and could be easily implemented early after stroke in the hopes of improving upper extremity outcomes. Given this, we believed this device could potentially be used for a trial of early robotic rehabilitation if appropriate tasks were designed. Therefore, our objective was to develop new approaches to augment recovery in the subacute phase of stroke.

We developed a set of rehabilitation tasks, in consultation with therapists, physicians and stroke survivors, for use in a pilot trial exploring the impact of robotic rehabilitation beginning in the subacute phase post-stroke. The purpose of the current manuscript is to review the development of stroke rehabilitation tasks for the Kinarm, assess its feasibility in a small cohort of participants, as well as to present the results of the pilot study in which they were tested. We expected that participants would attend and complete at least 80% of the robotic intervention sessions with few dropouts and adverse events. We further hypothesized that subacute stroke participants who received supplemental robotic therapy using the Kinarm, while continuing with standard of care therapy, would show greater improvements on standardized clinical measures compared to those who only received standard of care.

## Methods

### Rehabilitation device

The Kinarm Exoskeleton lab includes two planar exoskeleton robotic systems and an integrated virtual reality system [20] (Fig. 1; Kinarm, Kingston, Ontario). It supports free movement of the elbow and shoulder joints in the horizontal plane through the use of an adjustable four-bar linkage. Participants sit in a wheelchair base with their hands, forearms, and upper arms supported. Length and position of the linkages and troughs, respectively, can be adjusted to suit each participant. The device is then wheeled up to a horizontal virtual reality display. The participant’s arms remain under the display, with vision of their arms occluded by a metal shutter. A circular white cursor above the participant’s index finger on the visual display is commonly used to provide visual feedback of hand position during tasks. The movement of the participant’s elbows and shoulders can be monitored or manipulated by mechanical loads using encoders and torque motors.

### Rehabilitation Task Design

Based on previous literature, we incorporated the following 4 factors thought to positively influence stroke therapy in our rehabilitation tasks: (1) *Feedback*. This is the information provided to a participant about their performance on a task [34] and an important component to learning. More specifically, the use of visual and haptic feedback during task practice seems to improve learning [35]. In stroke survivors, feedback given within therapy enhances motor performance compared to conventional therapy [36] and decreases length of hospital stay [37]. (2) *Intensity of therapy.* This is the number of repetitions completed within a given time frame, which often improves outcomes after stroke when compared to less intense therapy [7, 38]. (3) *Challenge*. This refers to the maintenance of task difficulty over time. In healthy individuals, it has been suggested that there is an ideal level of challenge in which learning is maximized [39]. Challenge of therapy is suggested in the current Canadian Stroke Best Practice Recommendations [40]. (4) *Engagement.* A recent study found a correlation between enjoyment of therapy and clinical gains made in chronic stroke [41], meaning that more engaging tasks were related to better outcomes. With these factors in mind, we wanted to create tasks that provided feedback to the participant, required numerous repetitions, could become more challenging over time, and were engaging.

Tasks were created to target different aspects of sensorimotor function. They incorporated the ability of the robot to carry out unimanual or bimanual tasks and provide forces for assistance and/or resistance. The robotic tasks aimed to increase range of motion, speed, and accuracy of movement, as well as improve the awareness of limb position and movement.

### Level design

With input from a physiatrist, physical therapists, and occupational therapists, levels were created to maintain task difficulty throughout the therapy program. This is discussed in more detail below. Preliminary testing was conducted with five individuals with subacute stroke and four healthy controls. These individuals provided qualitative feedback on the difficulty of each task level. This feedback helped ensure level progression followed a logical order. For example, when little difference in performance was noted between levels of a task, a level was removed. Alternatively, if the difficulty in moving from one level to the next was felt to be far too large, an intermediary level was implemented. Preliminary testing of these individuals also informed the performance cut-off required to move up a level, which is described for each individual task below. Level 1 was created for individuals with minimal functional ability of the affected limb. Design factors included: keeping the workspace of the task relatively small in size, making features of the task salient (e.g., large-sized targets), minimizing the speed of moving objects in the task so that they could be captured easily by an individual with lower function of the limb, and ensuring that any limb movements that were required by the task were short in distance and/or were facilitated by assistive forces. For subsequent levels we aimed to alter only one task element at a time. For example, to increase difficulty from level 1 to level 2, the size of the target was reduced, *or* the reaching distance was increased. Levels were modified to be more challenging in each subsequent level until a point was reached in which healthy young controls had consistently poor performance on the task. Each level was also designed with an achievement in mind in which the participant would move on to the next level. For example, in Assist/Resist, the participant would need to reach to 56 targets within 4 min before they would be moved on to the next level. All level setting was done prior to the pilot trial described below.

### The therapy tasks

**Fig. 2:**
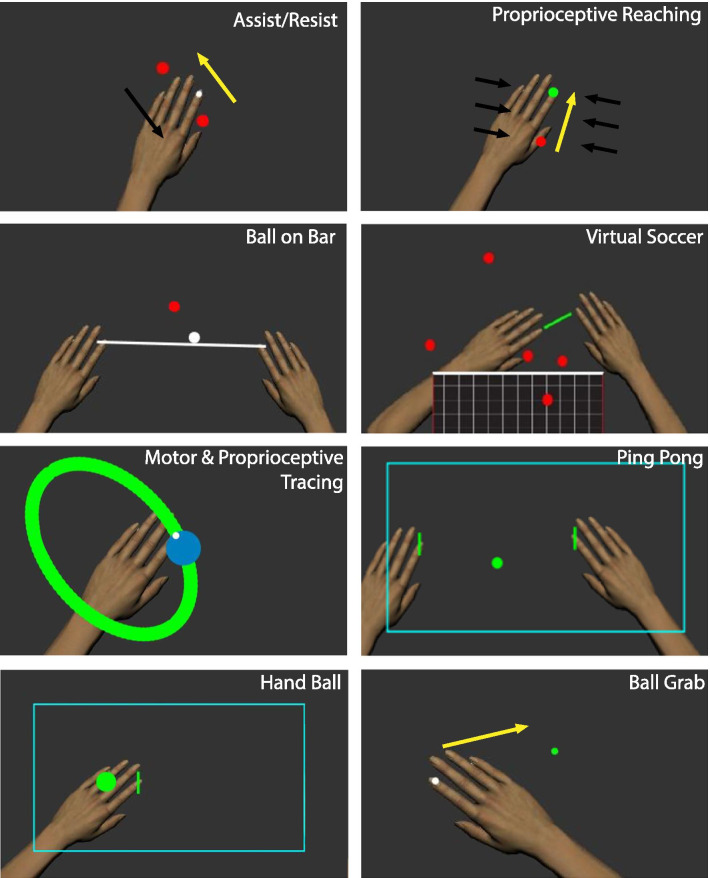
Visualization of robotic therapy tasks. Visual examples of some of the tasks used within the pilot study. In these tasks, and all other tasks, participants are not able to see their arms. These visuals have been added to assist in understanding how the subjects interact with the virtual environment. Yellow arrows indicate the direction of hand movement in the reaching tasks. Black arrows indicate the direction of the forces applied during the task, when applicable

For the pilot study, we created and tested 9 tasks for the Kinarm exoskeleton (Fig. 2). Descriptions of the therapeutic goals of each task and parameters that could be altered by the experimenter are in Table 1. Each task produced a score that allowed the robot operator to monitor participant improvement over time. Higher therapy task scores indicated better performance.

**Table 1:** Difficultly scaling parameters and functions targeted for robotic therapy tasks

Task	Increase difficulty	Functions targeted
Assist/resist	Reduce assistive force Increase resistive force	Strength Accuracy Speed Range of motion
Proprioceptive reaching	Reduce assistive force Reduce virtual radius size More challenging target locations Reduce target size	Sensation Speed Accuracy
Ball on bar	Increase ball movement Reduce ball friction	Bimanual coordination Speed Accuracy
Virtual soccer	Increase ball speed Change net location and size Reduce paddle size	Bimanual coordination Speed Accuracy
Motor tracing	Increase speed of chase target Increase acceleration of chase target Reduce size of chase target Make shape more complex Increase shape size	Motor control Accuracy
Proprioceptive tracing		Sensation Motor control Accuracy
Pong	Increasing possible ball speed Decrease ball size Increase ball elasticity Decrease paddle size Increase court size	Bimanual coordination Motor control Accuracy
Hand ball		Motor control Accuracy
Ball Grab	Decrease end target size Decrease amount of time for reach More challenging target locations	Speed Accuracy Range of motion

This table outlines how the nine tasks used as part of this pilot study increase in difficulty and which functions are targeted

*Assist/resist:* An out-and-back reaching task designed to improve movement speed, movement accuracy, and strength of the participant’s affected arm. Participants began at a central target and were instructed to move their index finger (represented by a white cursor) as quickly and accurately as possible to peripheral targets that would appear around the central target. They would then reach back to the central target and repeat the process. A total of 4 different peripheral targets, 10 cm away from the central target, were possible. These targets appeared in a block-randomized fashion and did not change location across levels. The lowest levels of this task employed assistive spring forces towards the target to aid in reaches. In higher levels, resistive forces away from the target were applied, to a maximum of 6 N, to oppose reaches. Participants completed 56 reaches over the course of 3–5 min. Participants were moved up to the next level if they could successfully complete the level within 4 min. A task score was calculated by multiplying the task level and the maximum velocity achieved by the participant averaged across each reach in that level.

*Proprioceptive reaching:* Similar to Assist/Resist, participants reached to different spatial targets using their affected arm, except vision of both arms was occluded. Targets were red circles, 2 cm in diameter. As soon as the subject left the starting position, the white cursor representing the subjects’ hand position disappeared, thus removing all visual feedback of limb position. A virtual radius existed around the end target that was not visible to the participant. When the participant reached within this radius, visual feedback returned, and the participant could finish reaching to the end target, which turned green upon the cursor entering the target. The size of the virtual radius was 3 cm on low levels but decreased as levels became more challenging, to a minimum of 1 cm. This task also employed assistive spring forces in early levels, to help guide the reach, that pulled stronger the further away a participant was from the target. The force started with a relatively strong spring and became progressively weaker for each subsequent level until the spring constant reached 0. Participants made anywhere from 72 to 120 reaches over 4–7 min. To move up to the next level, the participant had to complete levels 1–6 within 4 min and 45 s, and level 7 or higher within 6 min. A task score was calculated by multiplying the task level and number of targets successfully reached.

*Ball on bar:* This was a task initially designed to assess bimanual coordination [24]. Participant’s hands were connected visually by a horizontal bar on the display, and physically, using a strong spring force generated between the hands. A ball would appear on the bar and the participant had to move this ball, using the bar, to 4 possible targets that would appear in a set pattern on the screen. Early levels began with the ball fixed to the bar, but as levels progressed, the ball would move as the bar tilted and could roll off. If the ball rolled off the bar, it would reappear on the bar without consequence. Each level lasted for 1 min and the participant had to reach as many targets as possible during this time. The participant progressed to the next level when they successfully reached to at least 20 targets. A task score was calculated by multiplying the task level with the number of targets successfully reached.

*Virtual soccer:* This task was intended to improve bimanual and eye-hand coordination. Participants hands were connected visually and physically by a horizontal bar that acted as a paddle, similar to *Ball on Bar*. At the bottom of the screen, there was a soccer net that faced upwards. Balls would appear at the bottom of the screen and move away from the subject. The participant had to use their paddle to hit the balls back into the net. Counters for the number of balls hit and the number of goals scored were present at the top of the screen. Early levels use a large net (40 cm wide) and paddle (10 cm), but as levels progressed, the net and paddle would become smaller to a minimum size of 12 cm and 3 cm, respectively. An example of this task can be seen in Fig. 2. For each level, 300 balls moved across the screen, and the participant had to score at least 100 goals to progress to the next level. A task score was calculated by multiplying the task level and the number of goals scored.

*Motor tracing:* This task sought to improve movement accuracy and motor planning. Participants traced the outline of a shape with their affected hand. On the outline of the shape, a large red circle (the chase target) would appear, and the participant had to keep their fingertip cursor within this target, or at least touching it, as it moved around the outline. When the participant was within the chase target, it would turn blue. The percentage of time spent in the chase target was presented on the screen during the task. Early levels of this task used simple shapes like circles and ellipses that started off small, whereas harder levels used figure of eights and shapes that were larger. Early levels also used a larger chase target that traced slowly but this target became smaller and moved faster as levels became more difficult. For each level, the participant would trace 3 different shapes for 45 s each. Participants advanced when they maintained their fingertip cursor in the chase target for at least 80% of the time averaged across the 3 shapes. A task score was calculated by multiplying the task level and the percentage of time the participant spent within the chase target.

*Proprioceptive tracing:* This task sought to improve proprioception, movement accuracy and motor planning. It was similar to Motor Tracing, but visual feedback of the white cursor was intermittent, and vision of the arms were occluded. The chase target would still turn red when the participant’s cursor left the target and turn blue when the cursor was within the target. Early levels of this task would have the visual feedback of the cursor return for short intervals, whereas harder levels had longer intervals without visual feedback. For each level, participants would trace 3–4 different shapes for 45 s each. Participant advancement and task scores were identical to that of Motor Tracing.

*Hand ball:* This task focused on eye-hand coordination and motor planning. The participant had a vertical line (paddle) on their affected hand. A court would appear on the screen along with a ball inside of it. Inside the court, the participant had to make backhand motions to hit the ball against the wall on their affected side. They had to avoid letting the ball cross over to the other side of the court and hit the opposite wall. Counters for the number of times the ball hit the paddle or either wall were presented at the top of the screen. Ball hits to the top and bottom (horizontal) walls were inconsequential. Early levels of this task had a small court and used a large ball with low elasticity, but as levels progressed, the court became larger and the ball became smaller and more elastic. Each level of the task lasted for 2 min. Task performance was measured as the number of times the ball hit the wall on their affected side minus the number of hits to the opposite wall. To move to the next level, the participant had to exceed a task performance score of 40. Task score was calculated by multiplying the task level and the number of wall hits achieved by the more-affected hand.

*Ping pong:* A task designed to retrain bimanual coordination, eye-hand coordination and motor planning. This task was similar to Hand Ball except participants had to hit the ball back and forth between their hands. The participants had a vertical paddle representing each of their hands and had to hit the ball using forehand motions and prevent it from hitting the back wall on either side. The same counters for Hand Ball were present at the top of the screen and levels were progressed in a similar fashion. Each level lasted for 2 min. Task performance was measured as the number of times the participant hit the ball with the paddle on their affected hand minus the number of times the ball passed by and hit the wall. Individuals moved to the next level if they made approximately 40 successful hits with each hand. Task score was calculated by multiplying the task level and the number of wall hits achieved by the more-affected hand.

*Ball grab:* This task focused on improving movement speed. The participant moved their white cursor representing their fingertip to circular green targets, with a 2 cm diameter, that appeared in the workspace as quickly as they could. If they were unable to reach the target in time (within 5 s), it began to expand in size. The expanded target would then disappear when touched by the cursor. A countdown timer was present at the top of the screen as well as a counter for the number of targets hit. Targets that were hit after they began to expand were not counted as hits. In early levels of this task, targets remained close together, but as levels progressed, targets were gradually moved further away from the center of the workspace and became smaller (to a minimum size of 1 cm diameter), so the participant had to generate longer reaches. Each level lasted 200 s and the participant would need to reach to at least 100 balls in order to move on to the next level. Task score was calculated by multiplying the task level and number of targets successfully hit.

### Pilot study

We conducted a pilot study to evaluate the feasibility of a robotic training protocol in individuals with stroke. Participants completed 10 days of treatment (1 h/day) as part of the RESTORE (Robot Enhanced Therapy Optimizes REhabilitation) project.

### Participants

Potential participants with subacute stroke were identified by clinicians on the stroke units at the Foothills Medical Center and Dr. Vernon Fanning Care Center in Calgary, Alberta, Canada between January 2015 and July 2017. They were then approached by research staff about the trial to provide information about the study and enroll consenting individuals. Inclusion criteria were: age > 18 years, first stroke (ischemic or hemorrhagic), weakness or sensory loss noted in their medical chart, ability to follow two step commands, ability to speak and comprehend rudimentary English, corrected visual acuity of at least 20/50 with both eyes combined, and ability to commit to a 2-week long intervention with pre- and post-assessments. Exclusion criteria were: history of other neurologic disease(s) (ie. Multiple Sclerosis, Parkinson’s Disease), orthopedic issues limiting the upper extremity, and/or pre-stroke modified Rankin scale > 2. All participants provided informed consent.

### Sample size

A convenience sample was collected for the purposes of gaining sufficient numbers to obtain a preliminary idea of feasibility and feedback on our rehabilitation tasks. The pilot trial was stopped once we were convinced that the intervention was safe and feasible for the sample of subacute stroke survivors.

### Feasibility

For the purposes of this trial, we defined feasibility as the ability of the participants to attend and complete sessions, consideration of the number of participant dropouts, and the occurrence of adverse events. Further, we were specifically interested in whether the adverse events such as increased shoulder/arm pain, fatigue, deteriorating motor performance, and nausea would occur.

### Intervention

**Fig. 3:**
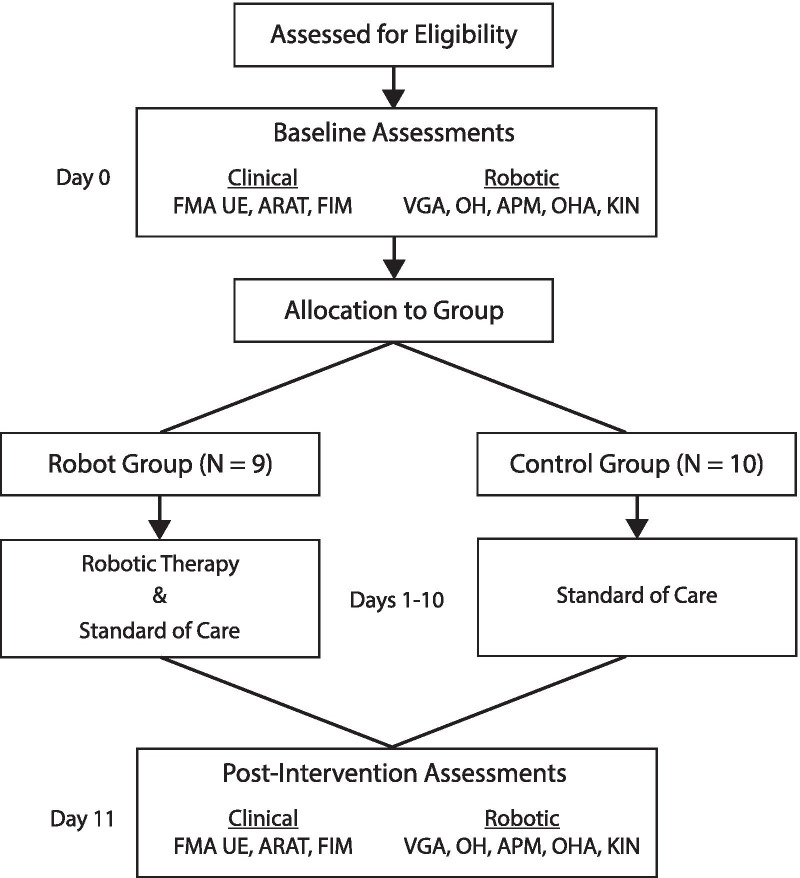
Study flow diagram. Participants in the robot group received 10 sessions of robotic therapy in addition to standard of care for 10 days, whereas those in the control group received only standard of care for 10 days prior to the follow-up assessments

Participants were allocated by research staff to receive either 1 h per day of robotic rehabilitation plus standard of care, or standard of care only using a matched-group design. This was done using baseline Fugl-Meyer Assessment of Upper Extremity (FMA UE) motor scores to roughly match subjects into each group. Standard of care therapy included an average of approximately 45 min of Occupational Therapy and 45 min of Physical Therapy per day [42]. For individuals in the robotic therapy group, the intervention occurred for 1 h/day for 10 days over two weeks and consisted of the nine robotic rehabilitation tasks, described above. A flow diagram of the study is in Fig. 3. Individuals in the intervention group also underwent a level setting session before starting robotic therapy to determine what was the appropriate level to start on for each task rather than having all individuals begin at level 1. This was done first by roughly estimating the appropriate level to begin training based on initial clinical evaluations and then adjusting levels based on performance on the robotic tasks. Every day, the tasks were completed in the same pseudo-random order for each participant, making sure to alternate between bimanual and unimanual tasks. Task order was different for each participant. A key objective was to keep the time spent on each task the same across days (about 6–7 min per task per session), although there was some variation due to participant performance.

In certain instances, participants were moved down a level from the previous day. This was done for a few reasons: the first of which was that some participants would ask to be moved down because they felt the current level was too difficult for them, regardless of their performance. Second, in some cases, participants could not reach all of the areas of the workspace required by a new level and were subsequently returned back to the previous level.

Baseline clinical and robotic assessments (described below) were collected prior to the beginning of the intervention. For the robotic therapy group, follow-up was done the day after completing the 10-day intervention, For the control group, follow-up assessments were collected 10 days after the baseline assessment (see Fig. 3).

### Clinical assessments

Clinical assessments included the FMA UE [43], to measure motor impairment of the upper limb and is scored from 0 to 66; the Action Research Arm Test (ARAT) [44], to measure upper extremity function on a scale from 0 to 57; and the Functional Independence Measure (FIM) [45], to measure functional abilities on a scale of 18–126.

### Robotic assessments

Alongside clinical assessments, participants also completed five standard robotic assessments, detailed below, that have all been previously described.

1. Visually Guided Reaching (VGR) is an 8-target center-out reaching task used to measure multi-joint movement, postural control, visuomotor response time, and coordination [22].
2. Object Hit (OH) is a bimanual object hitting task used to assess bimanual coordination [26].
3. Object Hit and Avoid (OHA) is a bimanual object hitting task that is used to assess bimanual coordination and higher-level cognitive function such as rapid decision making [21].
4. Arm Position Match (APM) is a position matching task used to assess proprioception, specifically limb position sense [23].
5. Kinesthesia (KIN) is a movement matching task used to assess proprioception, specifically sense of limb movement [25].

Participant performance was measured on a series of parameters specific to each task. A full list of the parameters measured for the first four assessment tasks is available online in the Kinarm Standard Task Summary [46]. The parameters for Kinesthesia are presented elsewhere [25]. Performance on each parameter was converted into a standard normal score based on performance of a large cohort healthy control subjects taking into account age, sex, and handedness on performance (Dexterit-E, version 3.4, Kinarm, Kingston, Canada). A global Task Score was also calculated from all normalized task parameters and transformed into a range where 0 indicated best possible performance and larger scores indicated worst performance (1.96 equalled 95th percentile for healthy controls). A more detailed calculation of these calculations can be found in the Kinam Standard Task Summary [46], see also [27].

### Analysis

Robotic therapy task scores were extracted from the data collected during therapy using custom scripts developed in MATLAB. Performance on therapy tasks at the beginning and end of the intervention were compared using a paired-samples t-test. An independent-samples t-test was used to compare baseline clinical and robotic assessment task scores between the control and robotic therapy group. Comparison of baseline and post-intervention clinical and robotic assessment scores within each group was done using a paired-samples t-test. All analyses were completed using IBM SPSS Statistics for Windows, version 26 [47].

## Results

Detailed participant demographics can be found in Table 2. The nineteen participants were, on average, 39.8 days post-stroke (SD = 20.50). Ten participants were in the standard of care group and nine were in the robotic therapy group.

**Table 2:** Demographics for study participants

Age	Sex	Handedness	Stroke	Affected side	Days Post	Pre FMA UE	Pre ARAT	Pre FIM	Post FMA UE	Post ARAT	Post FIM
*Robot therapy group*											
62	M	R	R VA	L	28	15	0	64	23	12	70
31	M	R	R PCA Hem	L	86	25	6	98	34	14	104
66	M	R	L MCA	R	24	63	53	79	64	53	113
43	M	L	L MCA	R	53	17	0	76	19	5	90
53	M	R	L PCA Hem	R	20	33	8	77	48	29	110
56	M	R	L MCA & ACA	R	58	13	3	86	15	4	103
64	F	R	R BA & PCA	L	69	46	53	120	52	56	121
35	M	R	R MCA	L	8	37	12	79	57	44	115
79	M	R	R MCA	L	39	40	20	85	50	32	86
*Control group*											
65	M	R	L MCA	R	9	65	55	111	64	57	121
68	M	R	R MCA	L	51	11	3	91	12	3	N/A
82	M	R	R MCA	L	28	12	3	86	11	0	103
50	M	L	L BA Hem	R	42	51	37	106	51	34	111
60	F	L	R MCA	L	62	9	3	69	9	3	N/A
26	M	R	L MCA	R	47	12	3	N/A	15	3	N/A
58	M	R	R BA	L	26	45	38	93	56	47	109
68	M	R	R PCA & L PICA	L	23	46	26	N/A	51	28	N/A
48	M	R	R MCA	L	36	52	29	111	50	40	116
52	M	R	R MCA	L	44	17	4	104	27	4	119

Demographic information for all participants included in this pilot study. All strokes were ischemic except when ‘Hem.’ is listed to indicate a hemorrhage. *BA*  basilar artery, *MCA*  middle cerebral artery, *PCA*  posterior cerebral artery, *PICA*  posterior inferior cerebellar artery, *VA*  vertebral artery

### Feasibility

Robot therapy was well-tolerated by the participants as every participant in the robotic therapy group was able to successfully complete the 1-h session each day of the intervention. Most reported they enjoyed the therapy tasks when asked by the study staff. Participants commonly reported feeling fatigued following the robotic therapy session, but no other adverse events occurred or were reported during the study, even with participants as early as 8 days post-stroke beginning the robotic intervention (Table 2). There were no dropouts in either group. A significant challenge faced with implementing robotic therapy was scheduling. Many participants had therapy schedules or medical tests, which were deemed a priority over the study by clinical staff and made organizing a consistent schedule over 2 weeks difficult.

### Performance on the robotic rehabilitation tasks

**Fig. 4:**
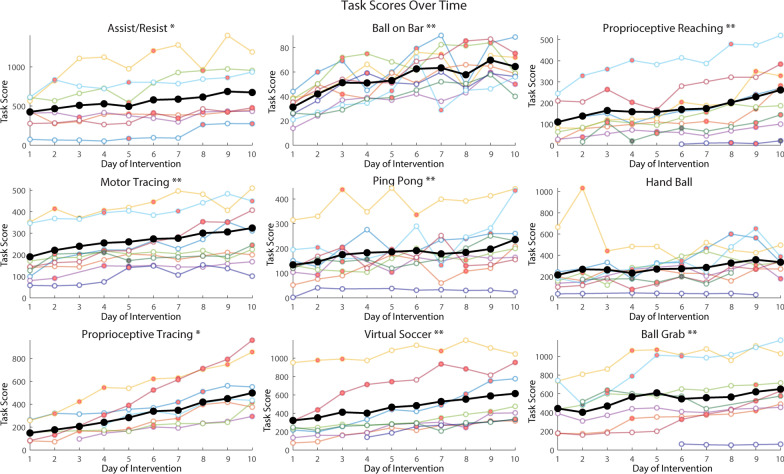
Robotic therapy task scores over time. The task scores of each participant over the course of the robotic intervention. Each panel indicates scores for a specific task. An open circle indicates that the participant completed the task on that day of the intervention. No circle is present if the participant did not complete the task on that specific day. Red inlays indicate a level-up on that day and black inlays indicate a level-down. The bold black line indicates average scores. Asterisks beside the task title indicates a significant improvement in score from day 1 to day 10. (* = p < 0.05 ** = p < 0.01)

We observed consistent improvement on the robotic therapy tasks across days (Fig. 4). Using a paired-samples t-test, we observed a significant increase in task scores from day 1 to day 10 of the intervention in 8 of the 9 therapy tasks including Assist/Resist, Ball on Bar, Proprioceptive Reaching, Motor Tracing, Ping Pong, Proprioceptive Tracing, Virtual Soccer, and Ball Grab. No significant change in task score was found in Hand Ball. While some of this improvement is due to the increase in task levels, one can see that scores tended to increase even when the same task level was used on sequential days.

### Clinical assessment outcomes

At baseline, the groups did not differ significantly for any of the clinical assessments. Following the intervention, we assessed the impact of the robotic therapy on clinical outcomes by comparing pre- and post-intervention clinical scores. The robotic therapy group achieved a significant increase across all clinical scores, with an 8-point increase noted for the FMA UE, a 10-point increase in ARAT, and a 16-point increase in FIM (Table 3). For the control group, no significant changes were found for the FMA UE or the ARAT, with only a 2.5-point and 2-point increase noted in each respectively. However, a significant increase was found for the FIM, with an increase of 11 points (Table 3).

**Table 3:** Comparison of pre and post clinical assessments within groups

Clinical assessment	Group	Pre-score Mean	Pre-score SD	Post-score Mean	Post-Score SD	P-value
FMA UE	Robot	32.11	16.47	40.22	17.90	0.005*
	Control	32.00	21.63	34.60	21.76	0.111
ARAT	Robot	17.22	21.22	27.67	20.12	0.017*
	Control	20.10	19.34	21.90	21.72	0.253
FIM	Robot	84.89	16.02	101.33	16.36	0.019*
	Control	96.38	14.52	113.17	6.77	0.004*

Results of the paired-samples t-test comparing the baseline and post-intervention FMA UE, ARAT, and FIM scores within the robotic therapy group and the control group. Starred groups indicate a significant improvement in the score

### Robotic assessment outcomes

At baseline, no significant differences were observed between the groups across all robotic assessments. The robotic therapy group demonstrated a significant improvement in Visually Guided Reaching from pre- to post-assessment. Significant improvement was not observed on any other robotic assessment (Table 4). In the control group, a significant improvement was observed in Arm Position Match from pre- to post-assessment, but not on any other robotic assessment (Table 4).

**Table 4:** Comparison of pre and post robotic assessments within groups

Robotic assessment	Group	Pre-score Mean	Pre-score SD	Post-score Mean	Post-score SD	P-value
VGR	Robot	6.45	2.84	3.69	2.67	0.002*
	Control	7.99	3.18	7.33	3.61	0.133
OH	Robot	4.57	2.53	4.19	2.69	0.593
	Control	5.62	2.61	5.30	1.95	0.607
APM	Robot	3.02	1.54	2.58	1.66	0.199
	Control	4.02	0.80	3.44	1.36	0.041*
OHA	Robot	4.43	2.62	3.33	2.33	0.153
	Control	5.91	2.26	5.02	1.73	0.151
KIN	Robot	2.70	1.23	2.34	1.21	0.068
	Control	3.69	1.09	3.76	1.82	0.875

Results of the paired-samples t-test comparing the baseline and post-intervention robotic assessment scores within the robotic therapy group and the control group. Lower scores represent better performance. Starred groups indicate a significant improvement in the score

## Discussion

Our findings from this pilot study suggest that the use of the Kinarm Exoskeleton for rehabilitation in subacute stroke is feasible with few adverse events occurring, none of which were serious or prevented participant continuation in the intervention. Over the 10-day intervention, we observed that participants in the robotic therapy group made consistent improvements on the rehabilitation tasks. Performance on almost all robotic rehabilitation tasks was significantly better at the end of the intervention compared to baseline. Our robotic intervention group also displayed significantly higher FMA UE, ARAT, and FIM scores at the end of the pilot study compared to baseline. The intervention group also demonstrated a significant improvement on Visually Guided Reaching, possibly because of the similarity between the assessment and our robotic rehabilitation tasks. These findings suggest that the newly designed robotic therapy tasks for the Kinarm could potentially improve some clinical outcomes after stroke, but our results must be interpreted with obvious caution given this was a pilot study.

Notably, our control group made minimal improvements on the FMA UE, ARAT, and all robotic assessments except the Arm Position Matching task. Based on other studies [48, 49], we might have expected much larger gains in the control group in the clinical measures, but these did not materialize. This may be due to difference in the standard of care provided to the control group in our study compared to other studies. That said, our control group did improve significantly on the FIM, although this could potentially be due to compensatory behaviours.

The findings from this study agree with previously published literature that suggests upper-extremity robotic therapy is safe and can be beneficial to clinical outcomes after stroke [50, 51]. However, it is important to understand how the tasks used in the present study compare to other upper-extremity robotic rehabilitation tasks. Several previous studies have used out-and-back or point-to-point reaching tasks [16, 52–56]. These types of tasks can be helpful but are relatively simple and focus solely on reaching behaviour. In the present study, participants completed three different types of upper limb reaching tasks: Assist/Resist, Proprioceptive Reaching, and Ball Grab. Proprioceptive Reaching adds novelty as a robotic rehabilitation task by requiring participants to rely more heavily on guiding their movements using proprioceptive feedback, rather than visual feedback.

Another less commonly employed robotic rehabilitation task in past studies, is tracing or tracking [57, 58]. Unlike pure reaching tasks, these require continuous upper limb movements and tracking of a moving target or goal. The present study also makes use of tracking/tracing tasks as seen with Motor Tracing and Proprioceptive Tracing. While similar to the tasks employed by Sanguineti et al. (2009) [57], our Proprioceptive Tracing does not entirely eliminate visual feedback through a blindfold, rather, it uses diminishing intermittent visual feedback to assist the subject over the course of the intervention. It remains to be seen whether there are any benefits of one approach over the other.

Presently, there are only a few bimanual robotic therapy tasks [57, 59, 60] that have been previously described in detail and a smaller number of studies that discuss the use of bimanual as compared to unimanual robotic therapy [61–68]. This partially reflects that many robotic rehabilitation platforms are designed to only engage the affected limb [11, 69–71]. The Kinarm, as a bimanual robot, is capable of running bimanual tasks, like Ball on Bar, Ping Pong, and Virtual Soccer. Pong-like bimanual therapy tasks have been previously used in other studies [60]. Virtual Soccer, as well as Hand Ball, seem to fall under the category of game-like therapy tasks. Other studies have employed game-like tasks for stroke therapy [60, 72], with a similar ‘hand ball’ task used in Simkins et al. (2013) [60]. It is clear that many tasks used in the present study share similarities with other robotic rehabilitation tasks, but the present study presents some unique tasks (Ball on Bar, Virtual Soccer) that have not been discussed in previous literature.

Robotic therapy also provides a unique opportunity to target functions that are challenging to address in conventional stroke therapy programs. Many stroke survivors display deficits of proprioception after their stroke [73], yet therapies to rehabilitate proprioception are limited in traditional settings [74, 75]. In the present study, tasks like Proprioceptive Reaching and Proprioceptive Tracing provide the ability to target proprioception. However, the utility and application of such a task requires further investigation. In the present study we did not see significant changes in our robotic assessments of proprioception and we wonder if this may be related to the actual dose of the intervention or because of the diversity of tasks employed, causing less time to be spent on each of these tasks individually.

For the purposes of this pilot study, we were interested in developing and testing a variety of task types. Our ultimate goal is to be able to tailor robotic rehabilitation sessions to an individual, utilizing only a subset of the tasks for each subject to target their specific impairments. For example, a stroke survivor with intact sensation but slowed movements could complete tasks that focus on improving movement speed rather than tasks that are more sensory based. Implementing this type of tailored robotic therapy would require further examination of the tasks employed so the individual effects of a single task could be disentangled from the entire intervention. Further, identification of when certain tasks should be employed to maximize potential recovery will need to be considered. Examining movement kinematics over the course of recovery may reveal that some tasks are best completed early versus later in recovery.

Beyond task design, variations in design of the robotic device employed (unimanual vs. bimanual, planar vs three-dimensional (3D), end-effector vs. exoskeleton) may have an impact on the outcome observed with an intervention. Head-to-head comparisons of robotic devices in stroke recovery are rare, and most that we are aware of have focused on the issue of unimanual vs. bimanual training [65]. Although the advantages of bimanual training have been touted by some [32, 33] and seem very plausible, studies to date comparing unimanual vs. bimanual have not necessarily demonstrated substantial advantages of bimanual training [65, 66, 68]. Although there has been some suggestion that 3D robots with greater degrees of freedom may hold advantages over planar devices, according to a review on a small number of studies [76], we are unaware of a study that compares both types of robots using similar tasks. Lastly, direct comparisons of upper-extremity end-effector and exoskeleton devices were very rare in our review of the literature [77]. In summary, more research is necessary to directly determine whether the theoretical advantages of certain design implementations actually result in the proposed benefits. In some cases, we particularly need to determine whether these benefits are worth the added cost and design complexity associated with them.

Designing and implementing a novel therapy program during the subacute phase after stroke comes with some challenges. With task design, we chose to design therapy tasks that were different from the robotic assessments, to avoid “teaching to the test”. In the end, we saw that participants improved on the training tasks, but not with many of the robotic assessments. Determining progression from one level to the next was also a challenge. In this study, this was done manually, meaning that the operator had to closely monitor participant performance to determine whether they met the threshold to move up to the next level. The use of an overall Task Score to monitor level progression presented some interesting challenges as well. In the present study, a metric of performance was determined for each individual task and was then scaled by the level of the task to generate a Task Score. We chose to approach task score in manner, as with increasing task difficulty, it is possible participant performance on a given kinematic parameter may degrade. For example, increasing resistance might reduce the participant’s maximum speed, even if they are making improvements in a more difficult task level. Therefore, scaling the Task Score using task levels allowed us to take this into consideration, but means that progression to a higher level is inherently linked to a higher Task Score, which may not be ideal.

Level design also brings out one of the limitations of the present study. Creation of levels for each task was intended to maintain task difficulty for the duration of the 10-day intervention. In designing these levels, we attempted to make systematic increases in task difficulty so that the challenge from one level to the next would remain continuous. However, previous studies have relied on automation of this process by using feedback controllers that increase difficulty automatically with improvement of participant performance [57, 78, 79]. In the future, automation of this process in our robotic therapy protocol would cut down on time, potential human error and increase training consistency.

Other limitations of the present study are the lack of a dose- or intensity-matched control group, randomization, or a measure of engagement. The robotic therapy group received the intervention on top of standard of care therapy, whereas the control group only received standard of care therapy. In essence, we were comparing groups who had received different amounts of therapy. This was strictly because our primary aim here was to examine the feasibility of using a new set of robotic therapy tasks in our participants with subacute stroke. As a preliminary study we also matched participants in our groups at baseline, rather than randomizing them. While randomization would have strengthened the study design, our study was only ever intended as a pilot to address feasibility before deciding to proceed with a larger, randomized trial to examine the efficacy of this therapy in subacute stroke.

## Conclusion and future directions

The above study demonstrated the feasibility of using our novel robotic rehabilitation program within subacute stroke. The participants were able to complete the entire intervention with some experiencing only minor issues with fatigue. Furthermore, the addition of out robotic therapy to standard of care results in significant improvement across clinical measures. Our next step is to conduct an appropriately powered, randomized study (RESTORE, ClinicalTrials.gov Identifier: NCT04201613) that will utilize an improved robotic intervention and clinical measures as a primary outcome to examine dose and timing. In the upcoming larger trial, we have chosen to double the number of days in which the participant will receive therapy, and some tasks, including Hand Ball, will be modified to better serve the participants based on our experiences with the present study. To assess dose and timing, multiple robotic therapy groups will be added to examine a potential interaction within this intervention. We also plan to add formal measurements of engagement. This Phase II Clinical Trial will see a substantial increase in the number of participants in order to appropriately power an analysis. Timing of participant recruitment will also be strictly limited to the first 5–9 days after stroke. This is important to point out as many clinical trials have previously focused on chronic stroke [1], so more studies that explore interventions early after stroke are needed. Ultimately, we postulate that the use of devices as an adjunct to existing standard of care therapy may be one way to augment recovery, particularly in situations where patients are receiving a limited amount of upper extremity therapy per day.

## Data Availability

Data available upon request.

## Abbreviations

FMA UE: Fugl-Meyer Assessment of Upper Extremity
ARAT: Action Research Arm Test
FIM: Functional Independence Measure
RESTORE: Robot Enhanced Therapy Optimizes REhabilitation

## References

[1] Cotoi A, Anderson A, Vermeer J, Al-Ibrahim F, McIntyre A, Teasell R (2017). Timing of motor rehabilitation after stroke: evidence from randomized controlled trials (RCTs). Int J Stroke.

[2] Ward NS, Brander F, Kelly K (2019). Intensive upper limb neurorehabilitation in chronic stroke: outcomes from the Queen Square programme. J Neurol Neurosurg Psychiatry.

[3] Daly JJ, McCabe JP, Holcomb J, Monkiewicz M, Gansen J, Pundik S (2019). Long-dose intensive therapy is necessary for strong, clinically significant, upper limb functional gains and retained gains in severe/moderate chronic stroke. Neurorehabil Neural Repair..

[4] Kunkel A, Kopp B, Müller G, Villringer K, Villringer A, Taub E (1999). Constraint-induced movement therapy for motor recovery in chronic stroke patients. Arch Phys Med Rehabil.

[5] Bernhardt J, Hayward KS, Kwakkel G, Ward NS, Wolf SL, Borschmann K (2017). Agreed definitions and a shared vision for new standards in stroke recovery research: The Stroke Recovery and Rehabilitation Roundtable taskforce. Int J Stroke.

[6] Masiero S, Poli P, Rosati G, Zanotto D, Iosa M, Paolucci S (2014). The value of robotic systems in stroke rehabilitation. Expert Rev Med Devices.

[7] Han C, Wang Q, Meng P, Qi M (2013). Effects of intensity of arm training on hemiplegic upper extremity motor recovery in stroke patients: a randomized controlled trial. Clin Rehabil.

[8] Winstein CJ, Rose DK, Tan SM, Lewthwaite R, Chui HC, Azen SP (2004). A randomized controlled comparison of upper-extremity rehabilitation strategies in acute stroke: a pilot study of immediate and long-term outcomes. Arch Phys Med Rehabil.

[9] Kwakkel G, Wagenaar RC, Twisk JWR, Lankhorst GJ, Koetsier JC (1999). Intensity of leg and arm training after primary middle-cerebral-artery stroke: a randomised trial. Lancet.

[10] Aisen ML, Krebs HI, Hogan N, McDowell F, Volpe BT (1997). The effect of robot-assisted therapy and rehabilitation training on motor recovery following stroke. Arch Neurol.

[11] Krebs HI, Hogan N, Aisen ML, Volpe B (1997). Robot-aided neurorehabilitation. IEEE Trans Rehabil Eng.

[12] Volpe BT, Krebs HI, Hogan N, Edelsteinn L, Diels CM, Aisen ML (1999). Robot training enhanced motor outcome in patients with stroke maintained over 3 years. Neurology.

[13] Masiero S, Armani M, Ferlini G, Rosati G, Rossi A (2014). Randomized trial of a robotic assistive device for the upper extremity during early inpatient stroke rehabilitation. Neurorehabil Neural Repair.

[14] Duret C, Gorsmarie A, Krebs HI (2019). Robot-assisted therapy in upper extremity hemiparesis: overview of an evidence-based approach. Front Neurol.

[15] Fasoli SE, Krebs HI, Hogan N (2004). Robotic technology and stroke rehabilitation: translating research into practice. Top Stroke Rehabil.

[16] Hogan N, Krebs HI (2004). Interactive robots for neuro-rehabilitation. Restor Neurol Neurosci.

[17] Veerbeek JM, Langbroek-Amersfoort AC, van Wegen EEH, Meskers CGM, Kwakkel G (2017). Effects of robot-assisted therapy for the upper limb after stroke: a systematic review and meta-analysis. Neurorehabil Neural Repair.

[18] Scott SH, Dukelow SP (2011). Potential of robots as next-generation technology for clinical assessment of neurological disorders and upper-limb therapy. J Rehabil Res Dev.

[19] Maciejasz P, Eschweiler J, Gerlach-Hahn K, Jansen-Troy A, Leonhardt S (2014). A survey on robotic devices for upper limb rehabilitation. J Neuroeng Rehabil.

[20] Scott SH (1999). Apparatus for measuring and perturbing shoulder and elbow joint positions and torques during reaching. J Neurosci.

[21] Bourke TC, Lowrey CR, Dukelow SP, Bagg SD, Norman KE, Scott SH (2016). A robot-based behavioural task to quantify impairments in rapid motor decisions and actions after stroke. J Neuroeng Rehabil.

[22] Coderre AM, Abou Zeid A, Dukelow SP, Demmer MJ, Moore KD, Demers MJ (2010). Assessment of upper-limb sensorimotor function of subacute stroke patients using visually guided reaching. Neurorehabil Neural Repair.

[23] Dukelow SP, Herter TM, Moore KD, Demers MJ, Glasgow JI, Bagg SD (2010). Quantitative assessment of limb position sense following stroke. Neurorehabil Neural Repair.

[24] Lowrey CR, Jackson CPT, Bagg SD, Dukelow SP, Scott SH (2014). A novel robotic task for assessing impairments in bimanual coordination post-stroke. Int J Phys Rehabil..

[25] Semrau JA, Herter TM, Scott SH, Dukelow SP (2013). Robotic identification of kinesthetic deficits after stroke. Stroke.

[26] Tyryshkin K, Coderre A, Glasgow JI, Herter TM, Bagg SD, Dukelow SP (2014). A robotic object hitting task to quantify sensorimotor impairments in participants with stroke. J Neuroeng Rehabil.

[27] Simmatis L, Krett J, Scott SH, Jin AY (2017). Robotic exoskeleton assessment of transient ischemic attack. PLoS ONE.

[28] Mang CS, Whitten TA, Cosh MS, Scott SH, Wiley JP, Debert CT (2019). Robotic assessment of motor, sensory, and cognitive function in acute sport-related concussion and recovery. J Neurotrauma.

[29] Logan LM, Semrau JA, Debert CT, Kenzie JM, Scott SH, Dukelow SP (2018). Using robotics to quantify impairments in sensorimotor ability, visuospatial attention, working memory, and executive function after traumatic brain injury. J Head Trauma Rehabil.

[30] Herter TM, Scott SH, Dukelow SP (2014). Systematic changes in position sense accompany normal aging across adulthood. J Neuroeng Rehabil.

[31] Semrau JA, Herter TM, Kenzie JM, Findlater SE, Scott SH, Dukelow SP (2017). Robotic characterization of ipsilesional motor function in subacute stroke. Neurorehabil Neural Repair.

[32] Kantak S, Jax S, Wittenberg G (2017). Bimanual coordination: a missing piece of arm rehabilitation after stroke. Res Neurol Neurosci.

[33] Sainburg RL, Good D, Przybyla A (2013). Bilateral synergy: a framework for post-stroke rehabilitation. J Neurol Transl Neurosci.

[34] van Vliet PM, Wulf G (2006). Extrinsic feedback for motor learning after stroke: what is the evidence?. Disabil Rehabil.

[35] Sigrist R, Rauter G, Riener R, Wolf P (2013). Augmented visual, auditory, haptic, and multimodal feedback in motor learning: a review. Psychon Bull Rev.

[36] Piron L, Turolla A, Agostini M, Succconi CS, Ventura L, Tonin P (2010). Motor learning principles for rehabilitation: a pilot randomized controlled study in poststroke patients. Neurorehabil Neural Repair.

[37] Teasell RW, Foley NC, Salter KL, Jutai JW (2008). A blueprint for transforming stroke rehabilitation care in Canada: the case for change. Arch Phys Med Rehabil.

[38] Harris JE, Eng JJ, Miller WC, Dawson AS (2009). A self-administered graded repetitive arm supplementary program (GRASP) improves arm function during inpatient stroke rehabilitation. Stroke.

[39] Guadagnoli MA, Lee TD (2004). Challenge point: a framework for conceptualizing the effects of various practice conditions in motor learning. J Mot Behav.

[40] Teasell R, Salbach N, Foley N, Mountain A, Cameron J, de Jong A (2020). Canadian stroke best practice recommendations: rehabilitation, recovery and community participation following stroke. Part One: Rehabilitation and Recovery following Stroke; 6th Edition Update 2019. Int J Stroke..

[41] Putrino D, Zanders H, Hamilton T, Rykman A, Lee P, Edwards DJ (2017). Patient engagement is related to impairment reduction during digital game-based therapy in stroke. Games Health J.

[42] Metzler MJ, Lindsay S, Shi J, Reglin P, Bagg A, Dukelow S (2012). Intensity in tertiary stroke rehabilitation: a quality assurance study. Stroke.

[43] Fugl-Meyer AR, Jaasko L, Leyman I, Olsson S, Steglind S (1975). The post-stroke hemiplegic patient. 1. A method for evaluation of physical performance. Scand J Rehabil Med..

[44] Van der Lee JH, De Groot V, Beckerman H, Wagenaar RC, Lankhorst GJ, Bouter LM (2001). The intra- and interrater reliability of the action research arm test: a practical test of upper extremity in patients with stroke. Arch Phys Med Rehabil.

[45] Keith RA, Granger CV, Hamilton BB, Sherwin FS (1987). The functional independence measure: a new tool for rehabilitation. Adv Clin Rehabil.

[46] Kinarm User Guides and Documentation. https://kinarm.com/support/user-guides-documentation/. Accessed 24 June 2020.

[47] IBM Corp. Released 2019. IBM SPSS Statistics for Windows, Version 26.0. Armonk, NY: IBM Corp.

[48] Dromerick AW, Lang CE, Birkenmeier RL, Wagner JM, Miller JP, Videen TO, Powers WJ, Wolf SL, Edwards DF (2009). Very early constraint-induced movement during stroke rehabilitation (VECTORS): a single-center RCT. Neurology.

[49] Burgar CG, Lum PS, Scremin AME, Garber SL, Van der Loos HFM, Kenney D, Shor P (2011). Robot-assisted upper-limb therapy in acute rehabilitation setting following stroke: Department of Veterans Affairs multisite clinical trial. J Rehabil Res Dev.

[50] Mehrholz J, Pohl M, Platz T, Kugler J, Elsner B (2018). Electromechanical and robot-assisted arm training for improving activities of daily living, arm function, and arm muscle strength after stroke. Cochrane Database Syst Rev..

[51] Kwakkel G, Kollen BJ, Krebs HI (2008). Effects of robot-assisted therapy on upper limb recovery after stroke: a systematic review. Neurorehabil Neural Repair.

[52] Lo AC, Guarino PD, Richards LG, Haselkorn JK, Wittenberg GF, Federman DG (2010). Robot-assisted therapy for long-term upper-limb impairment after stroke. N Engl J Med.

[53] Pila O, Duret C, Laborne FX, Gracies JM, Bayle N, Hutin M (2017). Pattern of improvement in upper limb pointing task kinematics after a 3-month training program with robotic assistance in stroke. J Neuroeng Rehabil.

[54] Massie CL, Du Y, Conroy SS, Krebs HI, Wittenberg GF, Bever CT (2016). A clinically relevant method of analyzing continuous change in robotic upper extremity chronic stroke rehabilitation. Neurorehabil Neural Repair.

[55] Goffredo M, Mazzoleni S, Gison A, Infarinato F, Pournajaf S, Galafate D (2019). Kinematic parameters for tracking patient progress during upper limb robot-assisted rehabilitation: an observational study on subacute stroke subjects. Appl Bionics Biomech.

[56] Rodgers H, Bosomworth H, Krebs HI, van Wijck F, Howel D, Wilson N (2019). Robot assisted training for the upper limb after stroke (RATULS): a multicentre randomised controlled trial. Lancet.

[57] Sanguineti V, Casadio M, Vergaro E, Squeri V, Giannoni P, Morasso PG, Gaggioli A (2009). Robot therapy for stroke survivors: proprioceptive training and regulation of assistance. Advanced technologies in rehabilitation. Robots, wearable systems and brain–computer interfaces.

[58] Vergaro E, Casadio M, Squeri V, Giannoni P, Morasso P, Sanguineti V (2010). Self-adaptive robot training of stroke survivors for continuous tracking movements. J Neuroeng Rehabil.

[59] Squeri V, Cassadio M, Vergaro E, Giannoni P, Morasso P, Sanguineti V (2009). Bilateral robot therapy based on haptics and reinforcement learning: feasibility study of a new concept for treatment of patients after stroke. J Rehabil Med.

[60] Simkins M, Kim H, Abrams G, Byl N, Rosen J (2013). Robotic unilateral and bilateral upper-limb movement training for stroke survivors afflicted by chronic hemiparesis. IEEE Int Conf Rehabil Robot.

[61] Burgar CG, Lum PS, Shor PC, Van der Loos HFM (2000). Development of robots for rehabilitation therapy: the Palo Alto VA/Stanford experience. J Rehabil Res Dev.

[62] Lum PS, Burgar CG, Van der Loos M, Shor PC, Majmundar M, Yap R (2006). MIME robotic device for upper-limb neurorehabilitation in subacute stroke subjects: a follow-up study. J Rehabil Res Dev.

[63] Hesse S, Schulte-Tigges G, Konrad M, Bardeleben A, Wener C (2003). Robot-assisted arm trainer for the passive and active practice of bilateral forearm and wrist movements in hemiparetic subjects. Arch Phys Med Rehabil.

[64] Hsieh Y, Wu C, Wang W, Lin K, Chang K, Chen C (2016). Bilateral robotic priming before task-orientated approach in subacute stroke rehabilitation: a pilot randomized controlled trial. Clin Rehabil.

[65] Hung C, Hsieh Y, Wu C, Lin K, Lin J, Yeh L (2019). Comparative assessment of two robot-assisted therapies for the upper extremity in people with chronic stroke. Am J Occup Ther.

[66] Yang C, Lin K, Chen H, Wu C, Chen C (2012). Pilot comparative study of unilateral and bilateral robot-assisted training on upper-extremity performance in patients with stroke. Am J Occup Ther.

[67] Hung C, Lin K, Chang W, Huang W, Chang Y, Chen C (2019). Unilateral vs bilateral hybrid approaches for upper limb rehabilitation in chronic stroke: a randomized controlled trial. Arch Phys Med Rehabil.

[68] Wu C, Yang C, Chen M, Lin K, Wu L (2013). Unilateral versus bilateral robot-assisted rehabilitation on arm-trunk control and functions post stroke: randomized controlled trial. J Neuroeng Rehabil.

[69] Toth A, Fazekas G, Arz G, Jurak M, Horvath M. Passive robotic movement therapy of the spastic hemiparetic arm with REHAROB: report of the first clinical test and the follow-up system improvement. ICORR. 2005:127–30.

[70] Nef T, Mihelj M, Riener R (2007). ARMin: a robot for patient-cooperative arm therapy. Med Bio Eng Comput.

[71] Rosati G, Gallina P, Masiero S (2007). Design, implementation and clinical tests of a wire-based robot for neurorehabilitation. IEEE Trans Neural Syst Rehabil Eng.

[72] Nef T, Quinter G, Müller R, Riener R (2010). Effects of arm training with the robotic device ARMin I in chronic stroke: three single cases. Neurodegener Dis.

[73] Kenzie JM, Smerau JA, Hill MD, Scott SH, Dukelow SP (2017). A composite robotic-based measure of upper-limb proprioception. J Neuroeng Rehabil.

[74] Findlater SE, Dukelow SP (2017). Upper extremity proprioception after stroke: bridging the gap between neuroscience and rehabilitation. J Mot Behav.

[75] Carey LM, Matyas TA, Oke LE (1993). Sensory loss in stroke patients: effective training of tactile and proprioceptive discrimination. Arch Phys Med Rehabil.

[76] Kim G, Lim S, Kim H, Lee B, Seo S, Cho K (2017). Is robot-assisted therapy effective in upper extremity recovery in early stage stroke? A systematic literature review. J Phys Ther Sci.

[77] Lee SH, Park G, Cho DY, Kim HY, Lee J, Kim S (2020). Comparisons between end-effector and exoskeleton rehabilitation robotics regarding upper extremity function among chronic stroke patients with moderate-to-severe upper limb impairment. Sci Rep.

[78] Erol D, Mallapragada V, Sarkar N, Uswatte G, Taub E. Autonomously adapting robotic assistance for rehabilitation therapy. IEEE/RAS-EMBS International Conference on Biomedical Robotics and Biomechatronics, 2006;567–72.

[79] Borghese NA, Pirovano M, Lanzi PL, Wüest S, de Bruin ED (2013). Computational intelligence and game design for effective at-home stroke rehabilitation. Games Health J.

